# Branch Retinal Artery Occlusion as a presenting sign of Acute Retinal Necrosis: a rare association

**DOI:** 10.1186/s12348-020-0199-2

**Published:** 2020-02-12

**Authors:** Manisha Agarwal, Chanda Gupta, Abhishek Jain, Brajesh Kumar

**Affiliations:** Vitreo-retina Department, Dr Shroff’s Charity Eye Hospital, 5072, Kedarnath Road, Daryaganj, New Delhi, 110002 India

## Abstract

**Background:**

Acute retinal necrosis (ARN) is a potentially blinding necrotizing viral retinitis. It starts with one or more foci and spreads circumferentially and involves the posterior pole in the later stages. Vascular occlusions such as branch retinal artery occlusion, central retinal artery occlusion, and central retinal vein occlusion may occur secondary to underlying infectious etiology such as ARN.

**Findings:**

An elderly male patient with a history of coronary artery disease was diagnosed with branch retinal artery occlusion (BRAO) in the right eye and referred to the treating cardiologist. Few days later, he complained of diminution of vision in the left eye which made him seek another consultation when he was diagnosed to have ARN in the left eye, encroaching the posterior pole. He was investigated and treated for the same leading to minimal improvement of vision in the left eye possibly due to a delay in the starting of the anti-viral therapy.

**Conclusion:**

We report this case to highlight that occlusive vasculopathy can be a presenting sign of an underlying infectious etiology in any age group. BRAO was a rare presenting sign of ARN in our patient. A thorough peripheral examination is recommended in order to avoid missing infectious pathologies such as ARN which starts from the retinal periphery, progresses fast, and if not managed on time may lead to permanent loss of vision.

## Introduction

Acute retinal necrosis (ARN) is a potentially blinding necrotizing viral retinitis [1]. Vascular occlusions though rare may occur secondary to underlying infectious etiology such as ARN [2–4]. We report a case of an elderly male patient with a history of coronary artery disease who was diagnosed elsewhere with branch retinal artery occlusion (BRAO) in the right eye and referred to the treating cardiologist. An underlying ARN in the retinal periphery of the left eye was missed leading to a delay in the treatment and poor visual prognosis. This case highlights BRAO as a rare presenting sign of ARN.

## Case report

A 68-year-old male patient presented with sudden painless diminution of vision in the right eye 15 days before and gradual onset of blurring of vision in the left eye for the last 10 days. He had a systemic history of coronary artery disease, had undergone a cardiac bypass surgery 7 years before, and was on oral anti-hypertensives and anti-platelet drug (clopidogrel besilate 75 mg). He had an ophthalmic consultation 12 days before where he had been diagnosed to have BRAO in the right eye with a vision of counting fingers at 3 m, and the left eye was reported within normal limits with a vision of 6/6, N6. He had been referred to his treating cardiologist.

On examination, the best corrected visual acuity (BCVA) was finger counting at 3 m in the right eye and finger counting at 1 m in the left eye. Applanation tonometer recorded an intraocular pressure of 17 mmHg in the right eye and 8 mmHg in the left eye. Anterior segment examination of the right eye was within normal limits. The left eye showed keratic precipitates on the corneal endothelium, cells (grade + 2) [5] in the anterior chamber, and cells in the vitreous cavity (grade 2) [6]. The crystalline lens showed early cataractous changes in both eyes.

**Fig. 1:**
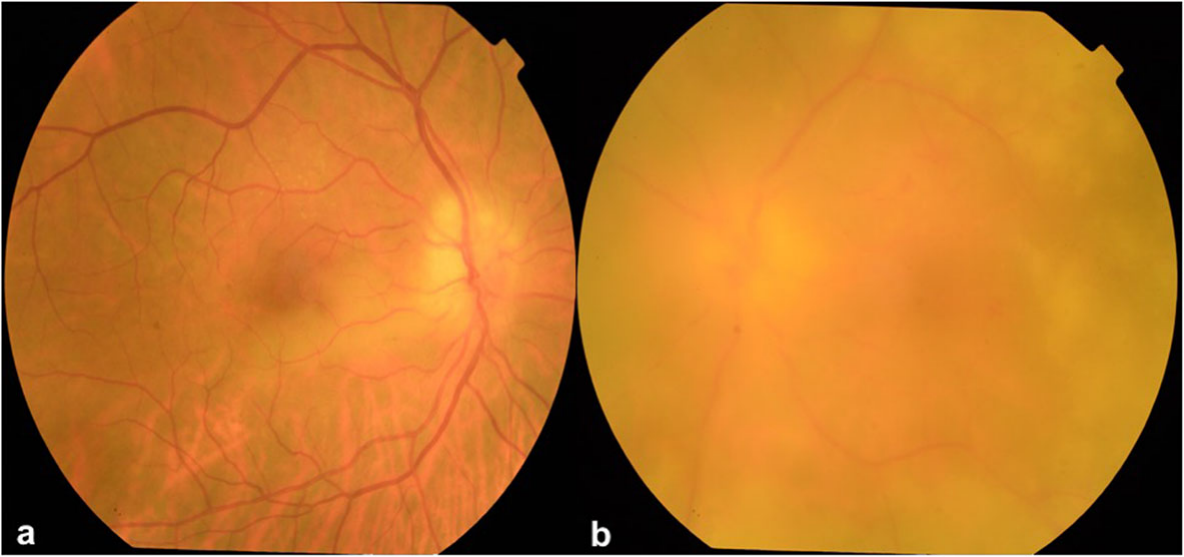
**a** Color fundus photograph of the right eye showing an area of retinal whitening inferonasal to the fovea and edematous disc with blurred margins. **b** Color fundus photograph of the left eye showing hazy media due to vitritis, edematous disc with extensive tongue-shaped whitish-yellow patches of necrotizing retinitis lesions outside the arcades encroaching the posterior pole

**Fig. 2:**
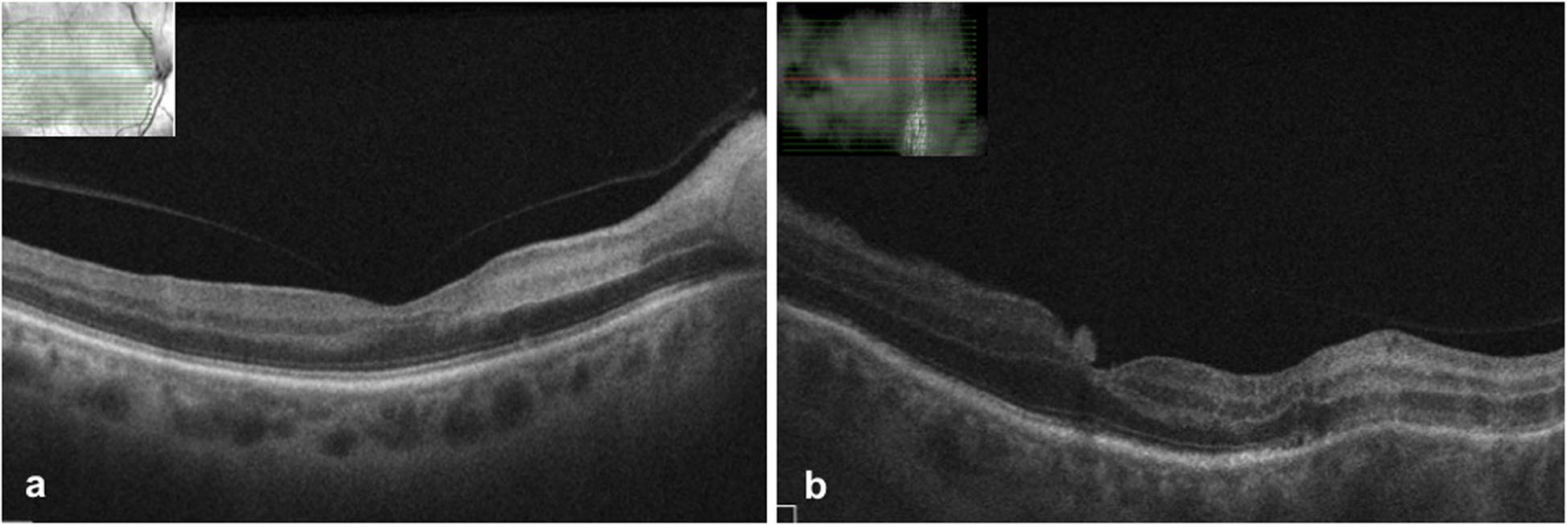
**a** OCT scan of the right eye showing hyper-reflective band in the inner plexiform and inner nuclear layers. **b** OCT scan of the left eye showing thickening of the retinal layers

**Fig. 3:**
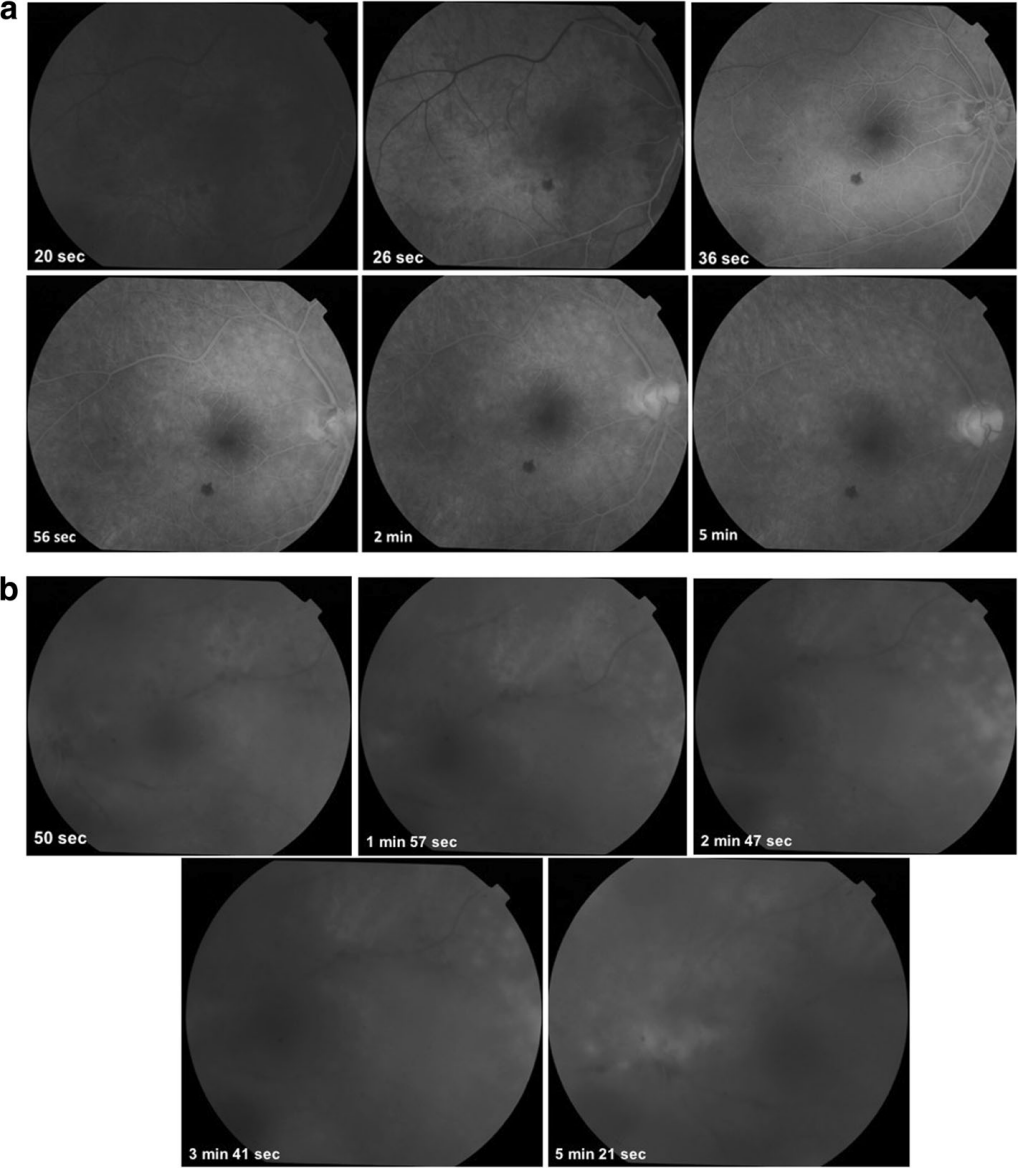
**a** Fundus fluorescein photographs of the right eye showing delayed arterial filling corresponding to the area of retinal edema. **b** Fundus fluorescein photographs of the left eye showing early patchy hyperfluorescence corresponding to the areas of retinitis and disc leakage in the late phase

Fundus examination of the right eye showed an edematous disc with blurred margins and an area of retinal whitening inferonasal to the fovea (Fig. 1a). Fundus examination of the left eye showed hazy media due to vitritis, edematous disc with extensive tongue-shaped whitish-yellow patches of necrotizing retinitis lesions outside the arcades encroaching the posterior pole (Fig. 1b). Optical coherence tomography (OCT) of the right eye showed a hyper-reflective band in the inner plexiform and inner nuclear layer nasal to the fovea, and the left eye showed mild thickening of the retina (Fig. 2a, b). Fundus fluorescein angiography (FFA) of the right eye showed delayed arterial filling corresponding to the area of retinal edema, and the left eye showed early patchy hyperfluorescence corresponding to the areas of retinitis and disc leakage in the late phase (Fig. 3a, b). A clinical diagnosis of BRAO in the right eye and ARN in the left eye was made.

Serological investigations showed raised cytomegalovirus IgG [107 U/ml (0–12 U/ml)] and IgM [21.8 U/ml (0–18 U/ml)] titers; IgG and IgM titers of herpes simplex virus and varicella zoster virus were within normal limits. HIV 1 and 2 were non-reactive. A presumed diagnosis of acute retinal necrosis due to cytomegalovirus infection was made. He was treated with six intravitreal injections of ganciclovir (2 mg/0.05 ml) and dexamethasone (400 μg/0.05 ml) twice a week in the left eye under strict aseptic precautions along with oral anti-viral therapy (valacyclovir 1 g three times a day). Oral corticosteroids 1 mg/kg body weight was started in tapering dosage 3 days after the initiation of the anti-viral therapy. By injection ganciclovir and dexamethasone, the viral infection and inflammation were addressed simultaneously.

**Fig. 4:**
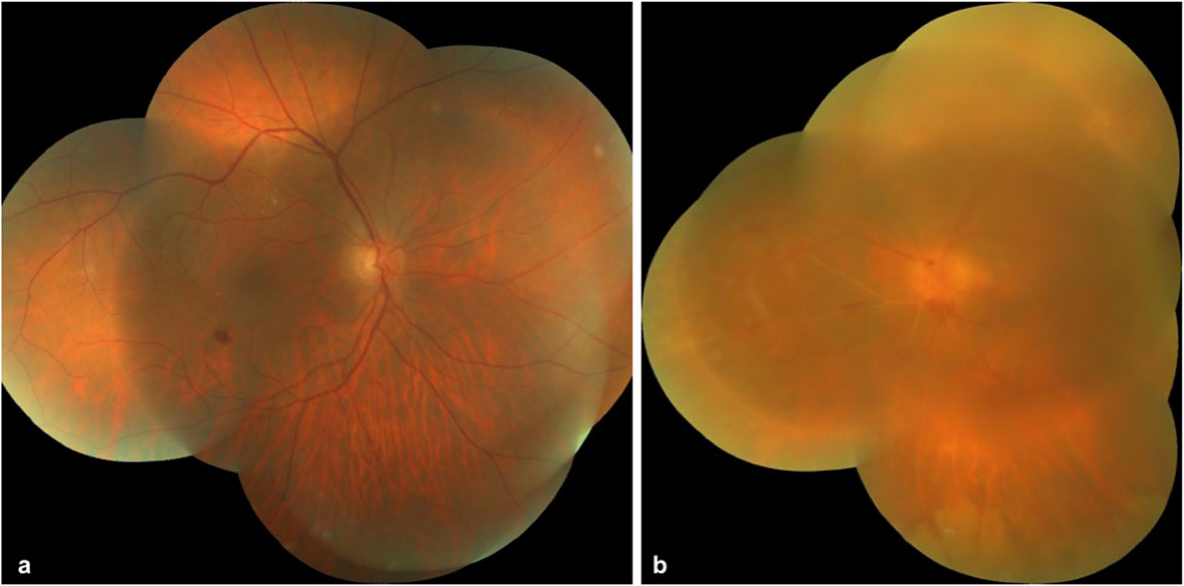
**a** Color fundus photograph of the right eye showing the resolved area of retinal whitening with reduced disc edema. **b** Color fundus photograph of the left eye showing reduced vitritis, resolving tongue-shaped necrotizing retinitis yellowish ill-defined lesions beyond the arcades, attenuated arteries, sclerosed vessels, and reduced disc edema

**Fig. 5:**
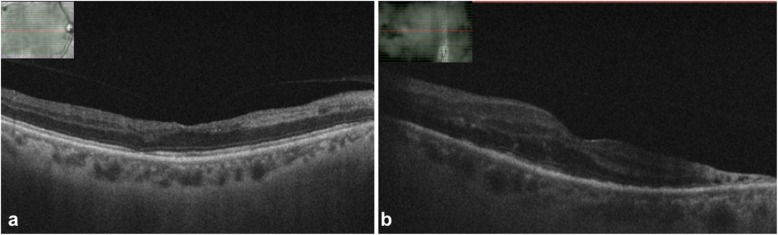
**a** OCT scan of the right eye showed thinning of the inner retinal layers. **b** OCT scan of the left eye showed diffuse cystic spaces and outer segment atrophy with ellipsoid (photoreceptors) loss

At 2 weeks follow-up, fundus examination of the right eye showed resolving retinal edema, and the left eye showed a reduction in vitritis, resolving disc edema and retinal lesions (Fig. 4a, b). At 1 month follow-up, BCVA in the right eye improved to 6/36 for distance and N24 for near vision, whereas BCVA in the left eye improved to finger counting at 3 m. Optical coherence tomography of the right eye showed thinning of the inner retinal layers, and the left eye showed thickening of the retinal layers (Fig. 5a, b). Prophylactic laser photocoagulation was done 360° posterior to the resolving retinitis patches in the left eye.

**Fig. 6:**
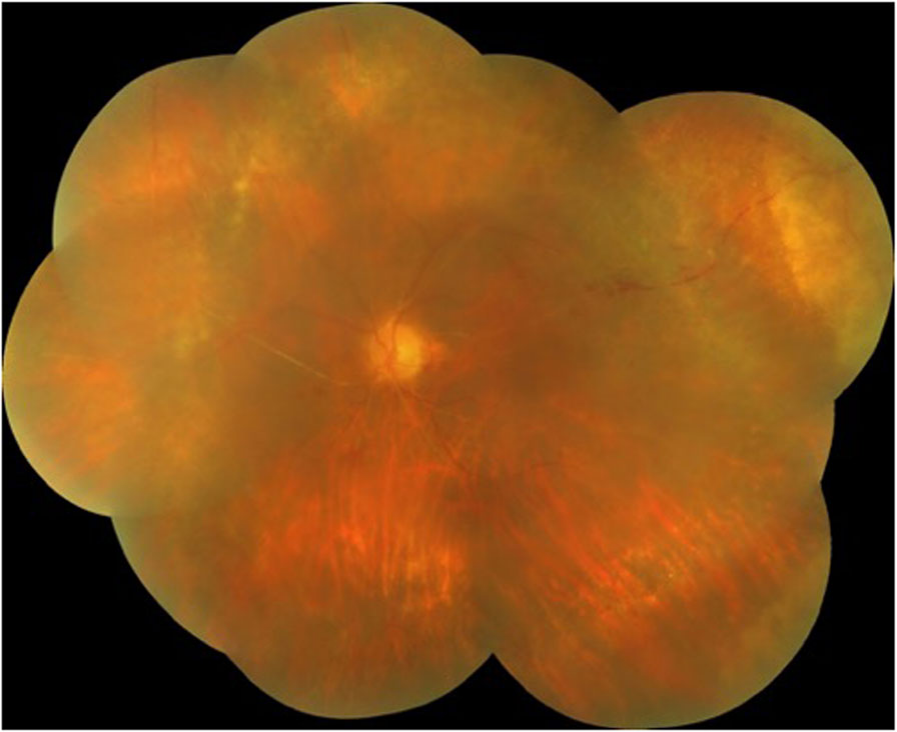
Color fundus photograph of the left eye showing pallor of the optic nerve head with sclerosed vessels, healed retinitis patches in the periphery, and chorio-retinal atrophy marks due to laser photocoagulation

At 3 months follow-up, BCVA in the right eye was maintained at 6/36 for distance and N24 for near and finger counting at 3 m in the left eye. Fundus examination of the right eye showed resolved retinal edema, and the left eye showed pallor of the optic nerve head with sclerosed vessels, healed retinitis patches in the periphery, and chorio-retinal atrophy marks due to laser photocoagulation (Fig. 6).

## Discussion

ARN is a rapidly progressive outer necrotizing retinitis usually sparing the posterior pole [3]. It starts with one or more foci and spreads circumferentially with associated evidence of occlusive vasculopathy [4]. Multiple members of the herpes virus family are known to be implicated in the pathogenesis of ARN such as varicella zoster virus (VZV), herpes simplex 1 and 2 (HSV-1, HSV-2), cytomegalovirus (CMV), and infrequently Epstein-Barr virus (EBV) [7]. The standard diagnostic criteria of ARN include clinical manifestations such as focal, well-demarcated areas of retinal necrosis located in the peripheral retina (outside the major vascular arcades); rapid, circumferential progression of necrosis with an evidence of occlusive vasculopathy; and a prominent inflammatory reaction in the vitreous and anterior chamber [8]. Histopathologically, there is a mononuclear cell infiltration with perivasculitis of both retinal and choroidal vessels. Doppler studies in such patients have shown the presence of a vascular compromise [9].

Heyreh, in a major critical review of 107 eyes of 78 patients with ARN, concluded that peripheral widespread retinal arteriolar occlusion and sheathing are the major vascular changes [10]. Duker et al. reported that branch retinal arterial or venous obstructive disease can manifest at any point in the clinical course of ARN [11]. Kang and Kim described a patient presenting with combined central retinal artery and vein occlusion in 1 eye who then subsequently developed ARN in the fellow eye [4]. Yokoi and Kase reported a case of vascular occlusion due to secondary syphilis [12].

The occlusion of a branch retinal arteriole may occur in any age group, though in the older age group, the most common cause is secondary to a thromboembolic phenomenon due to atherosclerosis [13]. In younger patients, it may occur secondary to infectious pathologies such as toxoplasmosis [14], CMV retinitis, HIV retinitis [15], Bechet’s disease, rickettsiosis, ocular tuberculosis, and West Nile fever [16]. It may also occur secondary to non-infectious causes such as IRVAN (idiopathic retinal vasculitis, aneurysms, and neuroretinitis) [16].

Our patient was an elderly male patient with coronary artery disease and a history of bypass surgery in the past, on anti-platelet drug. At his initial presentation with ophthalmic emergency of BRAO in the right eye to the first treating ophthalmologist, who keeping in mind his age and systemic history, referred him to his treating cardiologist to re-evaluate his cardiac status and look for a cause of the emboli. It was only when he started having a diminution of vision in the left eye due to the progression of the retinitis encroaching the posterior pole with enhanced inflammation that he presented to us and was diagnosed to have ARN. The possible mechanism for the occurrence of BRAO in the absence of any clinical evidence of inflammation in the right eye could be the presence of subclinical inflammation causing the release of inflammatory mediators which may disrupt the blood flow causing a thrombus formation leading to an arteriolar occlusion. However, as there was no evidence of active retinitis or inflammation in the right eye, the possibility of occurrence of BRAO unrelated to ARN cannot be excluded.

Although, raised titers of IgG and IgM for cytomegalovirus were noted, serological tests often show high false positivity and therefore are said to be less reliable for confirming the diagnosis. Polymerase chain reaction is a rapid and sensitive method for the detection of nucleic acids; however, our patient refused for any invasive test in his better-seeing eye. Though the CMV infection is rare in immunocompetent patients, it has been reported by Radwan et al. [17] and Tajunisah et al. [18] as a rare cause of ARN. Our patient though immunocompetent may possibly have an underlying relative immune deficiency as a result of immune senescence thereby making him at risk for CMV infection, and also the serological tests showed positive results for CMV, which made us presume the cause of ARN as a CMV infection.

The initial patches of retinitis might have been missed at the initial examination as they were limited to the extreme retinal periphery. This caused a delay in the initiation of the anti-viral therapy and impacted the final visual prognosis of the left eye.

We report this case to highlight the fact that occlusive vasculopathy might be the presenting sign of an underlying infective pathology in any age group. A thorough evaluation including the retinal periphery examination of both eyes is recommended in order to avoid missing infectious pathologies such as ARN which start in extreme retinal periphery as necrotizing retinitis but have a very fast progression towards the posterior pole causing a permanent loss of vision. Early diagnosis and timely management are of utmost importance in salvaging the vison in such eyes.

## Data Availability

All data generated or analyzed during this study are included in this published article.

## Abbreviations

ARN: Acute retinal necrosis
BCVA: Best corrected visual acuity
BRAO: Branch retinal artery occlusion
CMV: Cytomegalovirus
EBV: Epstein-Barr virus
FFA: Fundus fluorescein angiography
HIV: Human immunodeficiency virus
HSV: Herpes simplex virus
IRVAN: Idiopathic retinal vasculitis, aneurysms, and neuroretinitis
OCT: Optical coherence tomography
VZV: Varicella zoster virus
